# Anatomical and functional examination of superior colliculus projections to the inferior olivary in mice

**DOI:** 10.1007/s00429-025-03032-1

**Published:** 2025-12-08

**Authors:** Deviana David, Hugo Nusselder, Marylka Yoe Uusisaari

**Affiliations:** 1 Neuronal Rhythms in Movement unit, OIST, 1919-1 Tancha, Onna-son, 904-0495 Okinawa Japan; 2 Department of Neuroscience, Erasmus MC, Westzeedijk 353, Rotterdam, 10587 The Netherlands

**Keywords:** Superior colliculus, Inferior olive, Viral labeling, Immunohistochemistry, In-vivo calcium imaging, Optogenetic

## Abstract

The inferior olive (IO) is an important region for motor learning and movement coordination. IO activity carried by the climbing fiber (CF) projection to the Purkinje neurons in the cerebellar cortex drives the complex spike activity, central to theories of cerebellar function. Unlike many other neurons in the olivo-cerebeller system, IO neurons are not spontaneously active but rather spike in response to inputs from various regions of the brain. The superior colliculus (SC), a midbrain structure known for its role in orienting behaviors, is one of the input sources to the IO. Here, we investigate the SC projections to the IO using viral tracers, calcium imaging, and optogenetic stimulation. We reveal that, in addition to the known projections to the medial accessory olive (MAO), the SC axons also project to the ventral principal olive (PO). We show that SC axons terminate on both dendritic shafts and spines of IO neurons, potentially influencing not only spiking probability, but also the network synchronization mediated by gap junction coupling on dendritic spines. As a demonstration of the SC axons’ ability to drive IO spiking, we employ in vivo calcium imaging of the IO and show that optogenetic activation of SC inputs can drive spiking and modulate overall synchronization of the IO. This study provides a fundamental basis for studying the behavioral significance of the SC-IO pathway in mice.

**Supplementary Information:**

The online version contains supplementary material available at 10.1007/s00429-025-03032-1.

## Introduction

The inferior olive (IO) is part of the olivocerebellar system (OCS), which is important for motor learning and coordinated movement. The OCS consists of the interconnected networks of the IO, the cerebellar cortex, and the cerebellar nuclei (CN). The IO sends its axons, known as climbing fibers (CFs), to Purkinje neurons (PNs) in the cerebellar cortex. CF activity induces strong depolarization in the PN dendrites resulting in complex spikes (CSs), considered a key element of cerebellar function and motor learning (De Gruijl et al. 2013; Streng et al. 2018; Silva et al. 2024; Medina and Lisberger 2008; Wagner et al. 2021). Collaterals of the CFs from IO also target the CN, whose neurons form the final stage of cerebellar computation (Yarden-Rabinowitz and Yarom 2017).

Unlike the many types of spontaneous pacemaker cells in the OCS, spiking of the IO neurons is dependent on excitatory input. The afferents stemming from various regions of the brain converge on single cells (Ju et al. 2019), suggesting integrative processing within the IO. However, even though the combined effects of broadly-defined excitatory inputs and GABAergic feedback from the cerebellum have been investigated (Loyola et al. 2023), our understanding of the specific termination patterns of identified afferent pathways remains low.

Among its various sources of input, the IO is targeted by axons from the midbrain region called the superior colliculus (SC). The SC (known as the optic tectum in non-mammalian organisms; Isa et al. (2021)) is an evolutionarily conserved region that plays an important role in orienting movements and behavior (Zhou et al. 2023; Masullo et al. 2019; Solié et al. 2022; Basso and May 2017). In mice, the layered structure of the SC is considered to implement a spatial map of the external world, so that the anteroposterior (A-P) axis of the SC represents the front and rear visual fields, while the mediolateral axis of the SC represents the upper and lower visual fields (Cang et al. 2018; Ito and Feldheim 2018). The outputs of the SC diverge into numerous motor and nonmotor regions (Benavidez et al. 2021) that make further use of the information about the external world.

One of the less-investigated projections of the SC targets the IO. Studies across various species, such as macaques, cats, rats, and mice, have demonstrated that SC projects to the medial accessory olive (MAO; Kyuhou and Matsuzaki (1991); Akaike (1985); T. Hess D, (1982); May et al. (2023)). The fact that the neurons in the MAO project to the evolutionarily old paleocerebellum (cerebellar vermis; Sugihara and Shinoda (2004)) suggests that the tectal input could have a significant value as one of the first inputs to the IO (Northcutt 2002). Thus, the SC-IO connection can be seen as an excellent starting point to examine multisensory integration in the IO. However, the lack of detailed information on the precise location of SC-IO somata and the subcellular distribution of SC-IO synapses highlights the need for further anatomical investigations of this pathway.

Here, we complemented the investigation of the SC-IO pathway at mesoscopic and subcellular levels in mice. First, using anterograde and retrograde viral labeling, we mapped the SC-IO projection across the IO and SC regions. Surprisingly, we found that in addition to the previously known projection into the MAO, the principal olive (PO) also receives afferents from the SC. The PO-targeting SC neurons are intermingled with the MAO-targeting neurons in the deep and intermediate layers of the lateral SC.

We also examined the neurotransmitter identity of the SC-MAO and SC-PO axons, as well as identified the location of axonal terminals along the dendrites of IO neurons. We found that SC axons target dendritic shafts and spines that reside on distal dendrites, suggesting that activity in SC might not only drive spiking in IO neurons but also modulate levels of network synchronization that depend on electrotonic coupling via dendritic spines (Lefler et al. 2014; Leznik and Llinás 2005; de Zeeuw et al. 1998).

To demonstrate that the SC-PO pathway is capable of modulating olivary activity, we took advantage of the fact that the ventral fold of the PO, targeted by SC axons, is accessible for GRIN-lens-based imaging from the ventral side (Guo et al. 2021; Guo and Uusisaari 2025). Combining calcium imaging with targeted optogenetic activation of SC-IO axons confirmed that activation of the SC-IO pathway can evoke IO spikes as well as enhance network-level synchronization.

## Results

### Superior colliculus efferents target distinct regions in the principal and medial accessory olives

**Fig. 1:**
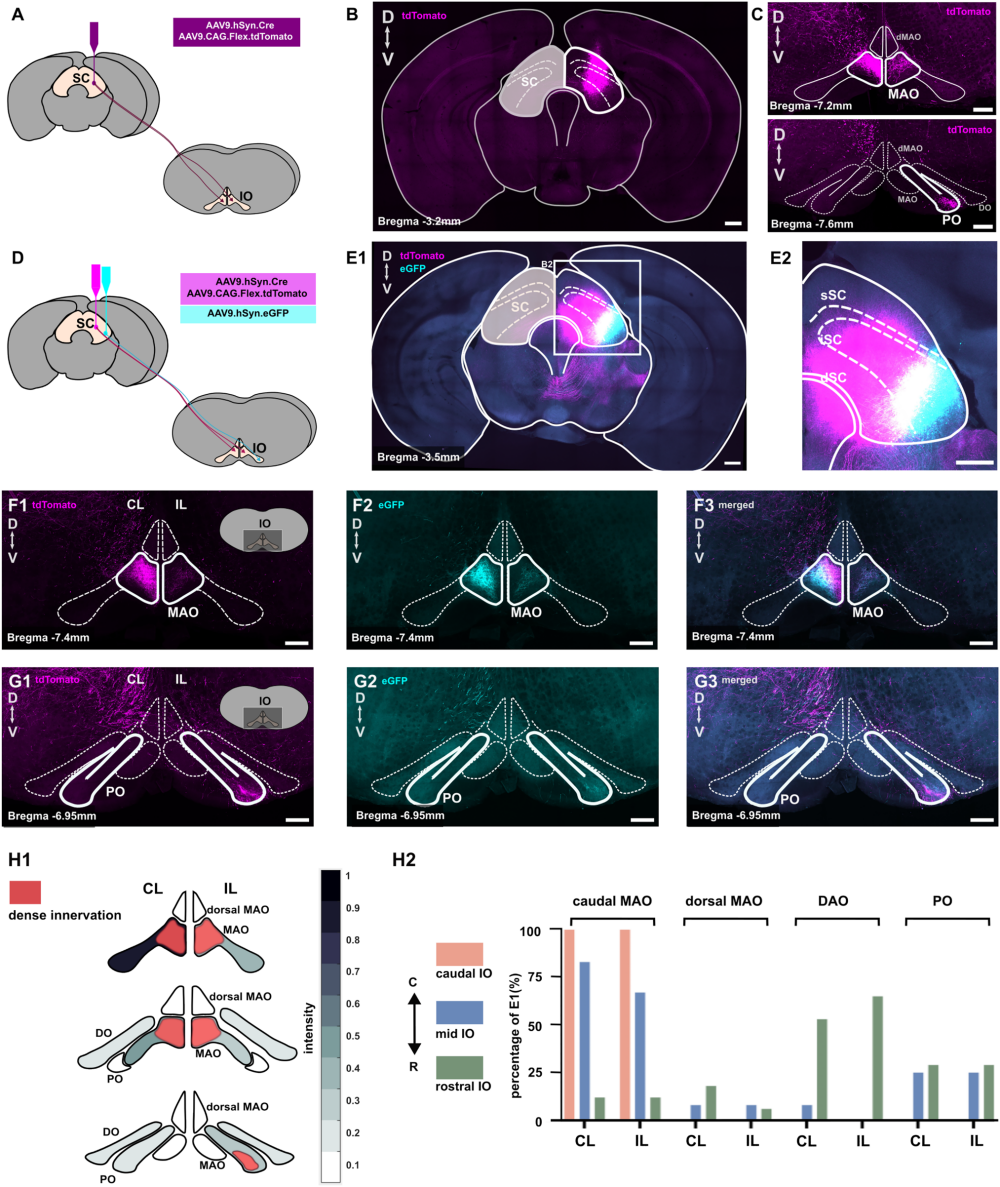
Superior colliculus (SC) projections to the medial accessory olive (MAO) and principal olive (PO). **A** Schematic of the injection of a mixture of AAV9.hSyn.Cre and AAV9.Flox.tdTomato to anterogradely label SC neurons, illustrated alongside a 10x confocal image of the midbrain and SC (**B**). Anterograde labeling reveals axons projecting not only to the MAO but also to the PO on the ipsilateral side (**C**). **D** Schematic showing the injection of a mixture of AAV9.hSyn.Cre, AAV9.Flox.tdTomato, and AAV9.hSyn.eGFP into different regions of the SC, resulting in labeled neurons within the medial and lateral SC, as depicted in (**E**). (**F1–3**) Images showing labeled SC axons in the contralateral and ipsilateral MAO, with another labeled axons found in the ipsilateral PO at the rostral part of the inferior olive (**G1–3**). (**H1**) Normalized density of labeled SC axons in different IO subnuclei along the rostro-caudal axis, with coordinates ranging from $-$6.9 mm to $-$7.9 mm from bregma. The red area indicates the termination zones of SC-IO axons. (**H2**) Probability distribution of finding labeled SC axons across different IO subnuclei, as shown in panel H1. Scale bar:: 200 $\mu$m.* CL* contralateral,* IL* ipsilateral

To visualize the IO regions targeted by the superior colliculus (SC), we anterogradely labeled the SC axons using a viral tracer driving the expression of the red fluorophore tdTomato (mixture of AAV9.hSyn.Cre and AAV9.CAG.Flex.tdTomato; Fig. 1 A-C). In addition to the well-established projection to the medial accessory olive (MAO), we discovered a restricted projection to the ventral fold of the principal olivary (PO) region. To explore the possibility that SC neurons projecting to MAO and PO originate from distinct SC regions, we employed two viral tracers tagged with different fluorophores, delivered into the medial and lateral regions of the SC (Fig. 1D). An example 10x confocal microscopy scan of the midbrain with labeled SC neurons in different medio-lateral (M-L) positions is shown in (Fig. 1E). Figures 1 E1-E2 show the expression of tdTomato-labeled neurons in the medial and lateral parts of the SC and the expression of eGFP in the lateral part of the SC, so that the lateralmost SC neurons were not double-labeled (indicated by the white region in Fig. 1E1-E2). The example 20x scan of the medulla containing the IO in Fig. 1 F1 shows SC axons labeled with tdTomato and GFP (Fig. 1 F2) in the contralateral subnucleus d of the caudal medial accessory olive (cdMAO). Here, tdTomato expression is seen closer to the midline, while eGFP expression is more toward the lateral part of cdMAO with a co-labeled area between (Fig. 1F3). Thus, medial SC neurons project to the medial part of the cdMAO while lateral SC neurons project to the lateral cdMAO. This SC-MAO projection aligns with the SC-IO projection pattern reported in previous findings (Benavidez et al. 2021). The ipsilateral projection of SC-MAO, albeit weaker than the contralateral projection, was consistently observed (Fig. 1F).

During careful examination of the SC-IO termination zones across all IO regions, we also found labeled axons in a small area corresponding to the ventral fold of the principal olive (PO). Figure 1G shows a 20x scan of the rostral medulla containing IO with labeled axons in the PO ipsilateral to the injection site (Fig. 1G1-G3).

Figure 1H1 summarizes our findings in 5 animals by combining the normalized average intensity of fluorescence labeling in 48 slices across the rostro-caudal axis of the IO. Most SC-IO axons were found in the caudomedial MAO and in the rostral ipsilateral PO. The highlighted area in red indicates the location of the densest projections into the MAO and PO. The percentage of regional observations with labeled SC-IO axons as shown in Fig. 1H1 in 5 animals is shown in the panel on the right (Fig. 1H2).

### Intermingling of superior colliculus neurons projecting to the medial and principal olive

**Fig. 2:**
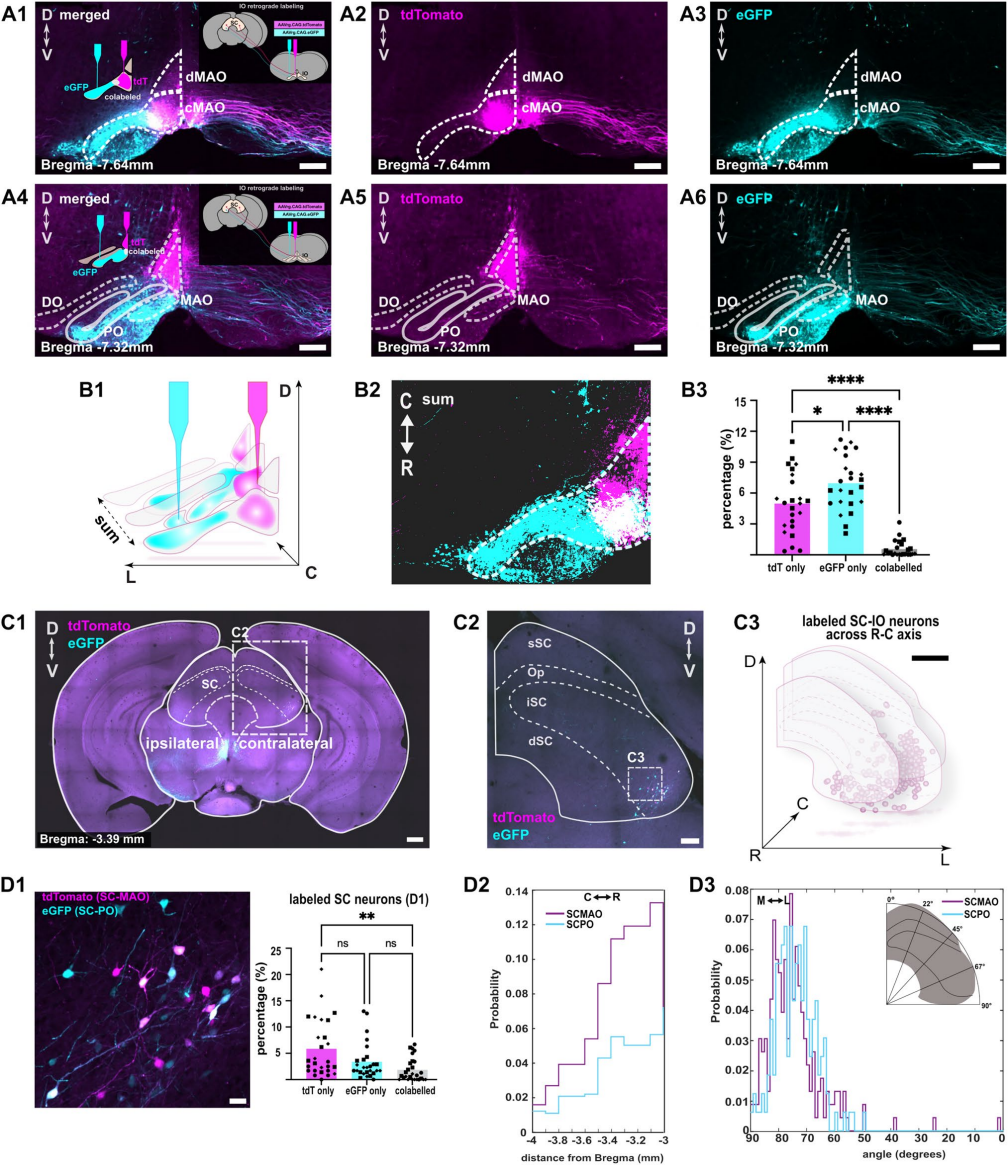
Identification of SC-MAO and SC-PO neurons via retrograde labeling from the inferior olive (IO). **A1** Confocal image showing merged channels of retrogradely labeled caudal MAO and PO following injections of AAVrg.CAG.tdTomato and AAVrg.CAG.eGFP, respectively. A schematic of the injection sites is shown in the upper left corner. **A2–A3** Same image as in A1, shown in separate color channels (tdTomato and eGFP). **A4** Merged confocal image of retrogradely labeled neurons in the more rostral IO, labeled with the same viruses as in A1. **A5–A6** Separate channels of the image shown in A4. **B1–B2** Summation of tdTomato-labeled (SC-MAO), eGFP-labeled (SC-PO), and co-labeled IO regions shown in panels A1–A6 across the rostrocaudal axis (n = 3;tdTomato vs eGFP ($P=0.01$), tdTomato vs colabelled ($P<0.001$), eGFP vs colabelled ($P<0.001$)). **(B3)** Quantification of the proportion of IO area labeled with tdTomato, eGFP, or both, corresponding to B1. **(C1)** Confocal image of a coronal midbrain section showing SC-IO neurons in the lateral intermediate layer of the SC, retrogradely labeled with tdTomato and eGFP. **(C2)** Images of SC and labeled SC-IO neurons with tdTomato and eGFP, respectively and summary of the location of the labeled SC-IO neurons across rostro-caudal axis (n=3, **(C3)**. **D1** Higher magnification images of labeled SC-IO neurons in **C2** and the quantification of SC neurons labeled with tdTomato, eGFP, or both across the rostro-caudal axis (n = 3; tdTomato vs eGFP ($P=0.062$), tdTomato vs colabelled ($P=0.001$), eGFP vs colabelled ($P=0.33$)). **D2** Stairs histogram showing the distribution of SC-MAO and SC-PO neurons along the rostrocaudal axis. **(D3)** Same data as in D2, plotted according to their angular coordinates. *C* caudal,* D* dorsal,* L* lateral

To provide additional support for the novel observation of SC projection into the PO as well as insights into the organization of the SC-IO pathway, we complemented the anterograde viral injections into the SC (Fig. 1) with dual retrograde viral injections into the medial and lateral parts of the IO (AAV9.hSyn.eGFP and mixture of AAV9.hSyn.Cre with AAV9.CAG.Flex.tdTomato); Fig. 2A) following our established protocol that allows accurate targeting with minimal spillage (Dorgans et al. 2022b, 2022). To address the challenge of confounding effects of possible spill-over of the virus between the PO and the MAO, and to avoid spread to the contralateral MAO, two retrograde viral vectors (one expressing tdTomato and the other eGFP) were injected into the MAO and PO on the same side within each animal. This approach improves confidence that retrogradely labeled SC cells with a single fluorophore (as opposed to expressing both tdTomato and eGFP) represent an exclusive projection to one of the target regions (Fig. 2A1,A4). Examples of IO confocal microscopy scans in which retrograde vectors were injected are shown for one mouse (out of 3) in Fig. 2A1-A6. Here, viruses expressing tdTomato and eGFP were injected into the medial and lateral regions of the IO, respectively. As expected, some regions of the MAO show co-labeling with both fluorophores, reflecting viral spread. However, the lateralmost IO region, including the PO, is exclusively labeled with eGFP. Therefore, we consider that SC neurons labeled only with eGFP project exclusively to the PO, whereas those labeled with tdTomato project to the MAO.

Figure panels 2 B1-B2, composed by summing the pixel intensities from 18 aligned image stacks (20x) from 100 $\mu$m sections from three animals. For each section, fluorescent signals from tdTomato and eGFP channels were converted into grayscale intensity matrices, manually aligned using midline and ventral surface of IO as references, and then summed on a pixel-by-pixel basis to generate cumulative heatmap of labeling density. The resulting composite revealed that only a small fraction of the IO was double-labeled (Fig. 2 B3) and the relative extents of the GFP and tdTomato labeling were consistent among the 3 animals, as quantified by the proportion of labeled pixels (tdTomato: 4.98 ± 0.60 $\%$; eGFP: 6.95 ± 0.53 $\%$; Fig. 2B3). The extent of double labeling was always minor (0.56 ± 0.16 $\%$).

Figure panels 2 C1-D1 show an example image of a region in SC with neurons retrogradely labeled with GFP or tdTomato, putatively targeting PO or MAO. The double-labeled SC neurons shown in Fig. 2D1 (indicated by white color) are likely to be the SC neurons that project to the colabeled area in MAO (Fig. 2A1). No labeled neurons were found in the superficial (sSC) and optic (Op) layers of SC (Fig. 2 C2-C3). Retrogradely labeled neurons were also observed in other regions of the midbrain such as the red nucleus and peri-aqueductal gray (PAG; See Supplementary Figure 01–02). Although a detailed investigation of midbrain sources of afferents in IO is beyond our current scope and means, the non-SC regions labeled in our samples were distant from the regions of SC investigated in this study, suggesting that the anterograde axonal labeling in the PO (as shown in Fig. 1 F-G) is not likely to be caused by unwanted viral spill-over into nearby regions. Even further support for the robustness of SC-PO projection was obtained by retrograde labeling of SC neurons following injection of Cholera Toxin Subunit B (CTB) conjugated to Alexa Fluor 555 into the lateral inferior olive, including the PO (see Supplementary Figure 03 A). Even though no CTB labeling was seen in the MAO region, CTB-labeled neurons were found in the lateral portions of the intermediate and deep layers of the superior colliculus (see Supplementary Figure 03B-C).

Next, we identified all neurons within the SC that were retrogradely labeled with tdTomato, eGFP, or both (Fig. 2 D1), where both PO- and MAO-projecting SC neurons are located. The majority of the retrogradely labeled neurons expressed exclusively tdTomato or GFP; overall, less than 5 $\%$ of them were double-labeled (n = 185 cells from 27 SC slices in 3 animals). Despite the fact that tdTomato labeling in the IO samples was less broadly than GFP labeling (Fig. 2B3), the majority of retrogradely labeled somata within the SC expressed tdTomato (Fig. 2D1), in line with the notion that the SC-MAO projection is stronger than the SC-PO projection. The SC neurons exclusively labeled with eGFP were always found intermingled with those exclusively labeled with tdTomato, even when the IO regions labeled with eGFP and tdTomato were clearly separated. As only a minority of neurons expressed both fluorophores (Fig. 2D1), it seems that divergence of SC projection to both IO regions is not common.

For quantitative assessment of the localization of SC-IO neurons projecting into different parts of the IO, we registered the labeled soma coordinates to reference space (Paxinos and Franklin 2001; see Methods). Indeed, in all animals, the SC-MAO and SC-PO neurons were intermingled with no apparent segregation along the rostro-caudal axis (Fig. 2D3) or the medio-lateral angular position (Fig. 2D2). Overall density of SC-IO neurons was higher in the rostral SC compared to the caudal SC (Fig. 2D1).

### Comparison of SC-MAO, SC-PO and non-IO-projecting SC neurons

**Fig. 3:**
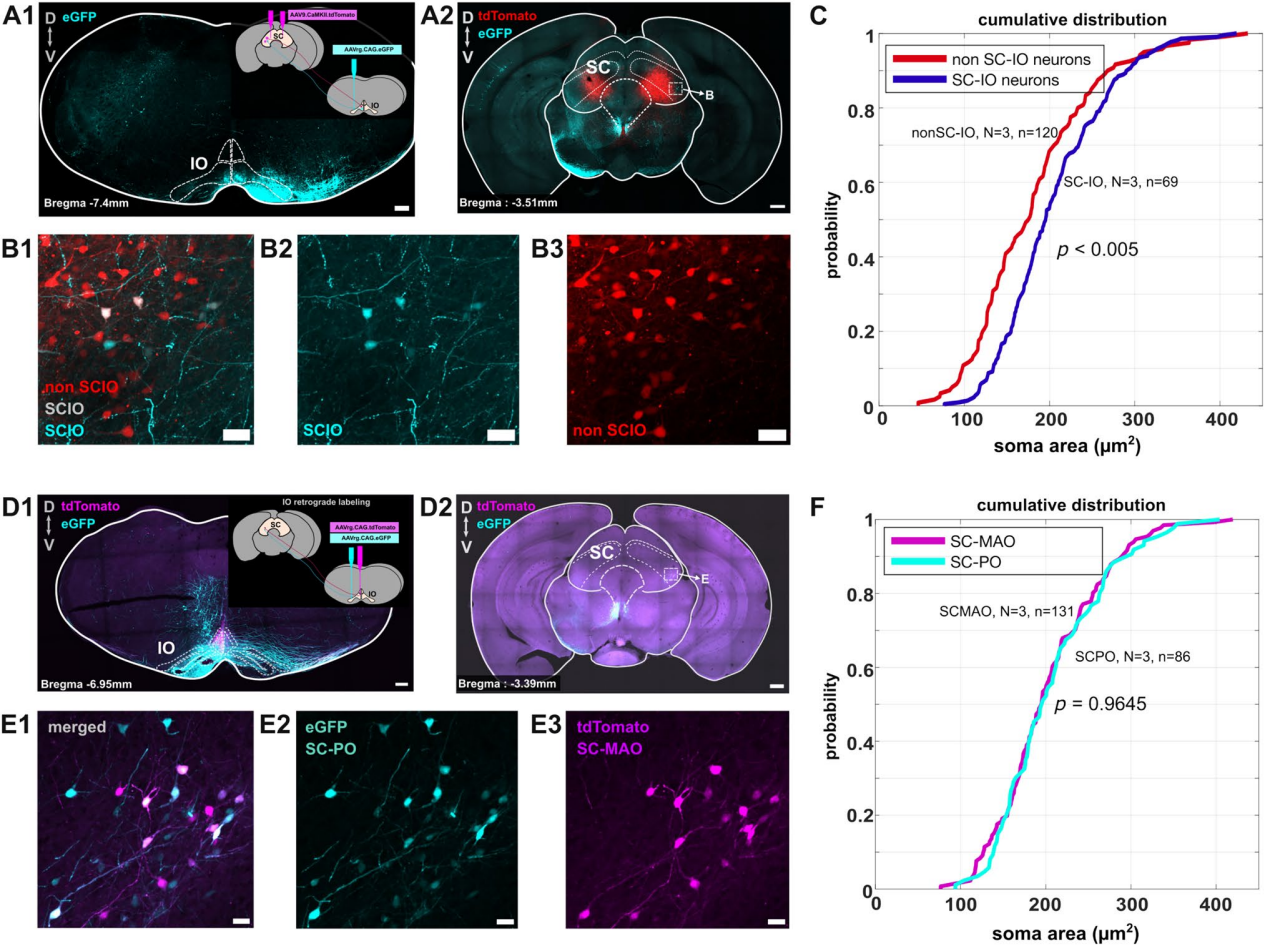
Comparison of SC-MAO, SC-PO and non-IO projecting SC soma size. **A1** Schematic illustrating the retrograde labeling of SC-IO neurons by injecting the AAVrg.CAG.eGFP into the IO and sparse labeling of SC neurons with AAV9.CAMKII.tdTomato. Scalebar: 200 $\mu$m (**A2**) Coronal section of the midbrain with the tdTomato labeled SC neurons. Scalebar: 200 $\mu$m (**B1–3**) a higher magnification of area indicated by square in the (**A2**), showing SC-IO neurons and non-IO projecting SC neurons. SC-IO neurons are indicated by the eGFP and colabeled neurons while non SC-IO neurons indicated by tdTomato-only labeled neurons. Scalebar: 20 $\mu$m. **C** Cumulative distribution of non SC-IO neurons and retrogradely labeled SC-IO neurons. Kolmogorov–Smirnov test shows SC-IO neurons are significantly bigger than non SC-IO neurons (Kolmogorov–Smirnov test, D = 0.31, ($P < 0.005$)).(**D1**) Schematic illustrating the retrograde labeling of different part of IO with AAVrg.CAG.eGFP labeling the lateral part of IO, including the PO and AAVrg.CAG.tdTomato labeling the medial part of IO as shown in Figure02. Scalebar: 200 $\mu$m. (**D2**) Coronal section of the midbrain with the superior colliculus and its retrogradely labeled neurons, showing tdTomato, eGFP and colabeled neurons. Scalebar: 200 $\mu$m. (**E1–3**) a higher magnification of area indicated by square in the (**D2**), showing colabeled neurons, eGFP and tdTomto labeled neurons. Scalebar: 20 $\mu$m. **F** Cumulative distribution of SC-MAO and SC-PO shows no significant differences between their soma sizes (Kolmogorov–Smirnov test, D = 0.068, ($P = 0.964$)). N indicates the number of animals, and n refers to the number of cells included in the analysis

Since no differences were found in the soma locations of the SC-MAO and SC-PO neurons, we further investigated whether these neurons could be distinguished by their soma sizes. For this purpose, we used standard deviation projection images from 20x confocal stacks of SC sections where SC-IO neurons were labeled with a single and somewhat larger-volume injection of retrograde virus into the IO. The other SC neurons were labeled with an additional viral reporter (mixture of AAV9.CamKII.Cre and AAV9.CAG.Flex.tdTomato) injected directly into the SC (Fig. 3A-B). In these images, we manually traced the broadest perimeters of the labeled somata to measure their cross-sectional areas.

Figure 3C shows the cumulative distribution between retrogradely labeled SC-IO and non-SC-IO (tdTomato-labeled) neurons. Statistical analysis using Kolmogorov–Smirnov test shows that SC-IO neurons were significantly larger than putative non-SC-IO neurons ($P= <0.005$, average SC-IO soma size 259.8 ± 9.23 $\mu$m^2^, average non SC-IO soma size 182.9 ± 5.22 $\mu$m^2^). We then compared the SC-MAO and SC-PO neurons. Figure 3F shows no differences in the cumulative distribution between SC-MAO and SC-PO ($P=0.96$). However, SC-PO neurons exhibited greater variability in size (average SC-MAO size: 193.7 ± 4.20 $\mu$m^2^; SC-PO: 203.4 ± 7.25 $\mu$m^2^).

### Immunohistochemistry of the SC-IO axons shows that majority of the boutons are glutamatergic

**Fig. 4:**
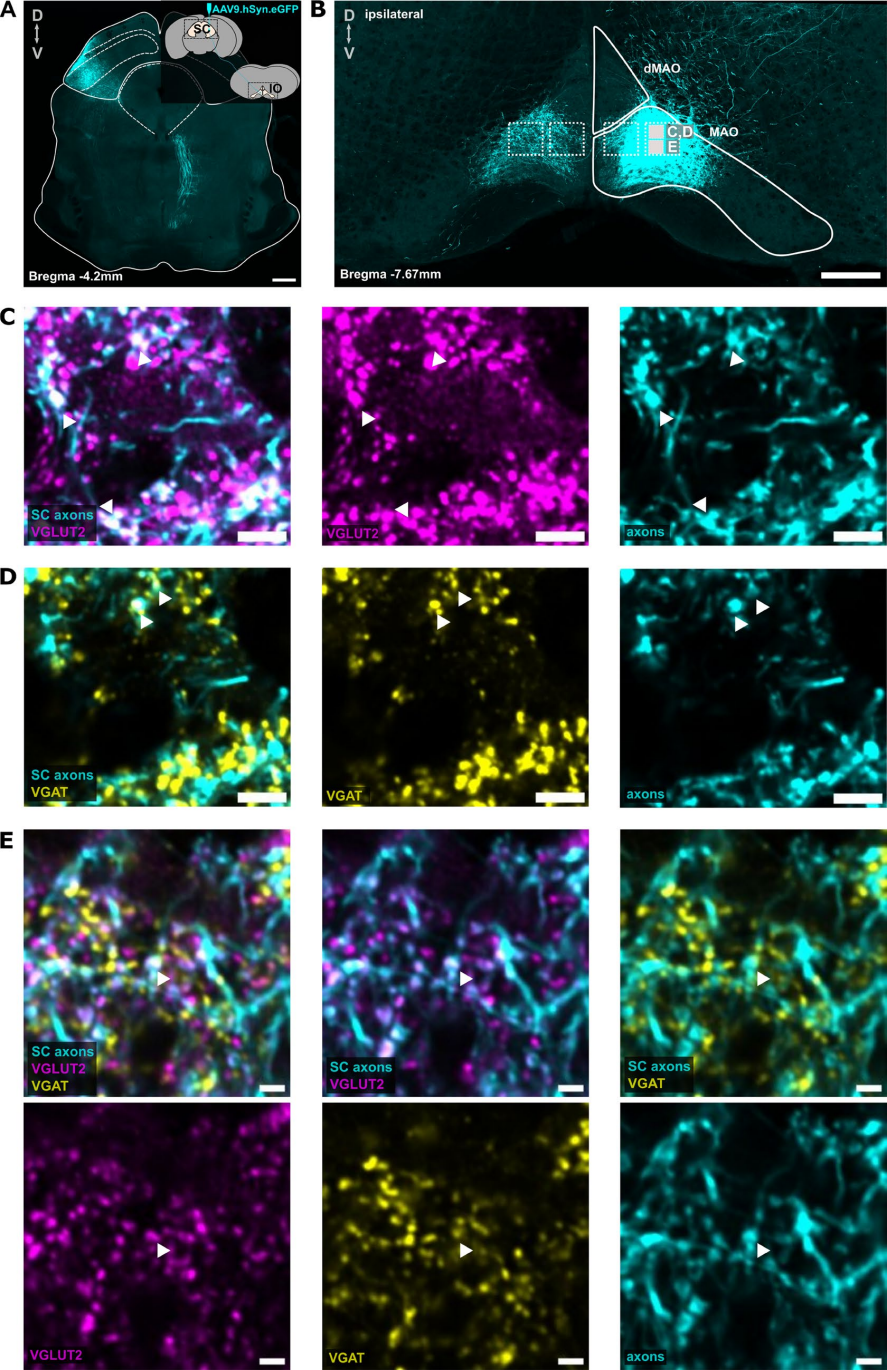
VGLUT2 and VGAT labeling in the medial accessory olive. **A** Anterograde labeling of SC neurons using AAV9.hSyn.eGFP, showing labeled SC axons in the medial accessory olive (MAO), as seen in **B**. The 100x100 $\mu$m squares indicate different regions of interest (ROIs) for 40x confocal acquisition of labeled VGLUT2, VGAT, and SC axons. **C** A 40X confocal acquisition of a section of MAO indicated by the squares in panel B, displaying labeled SC axons and VGLUT2, SC axons and VGAT (**D**), and SC axons with both VGLUT2 and VGAT **E**. White triangles in (**C-E**) indicate example boutons that were colocalized with VGLUT2, VGAT, or both. The scalebar of 200 $\mu$m for panel A, B and 5 $\mu$m for panel C-E

**Fig. 5:**
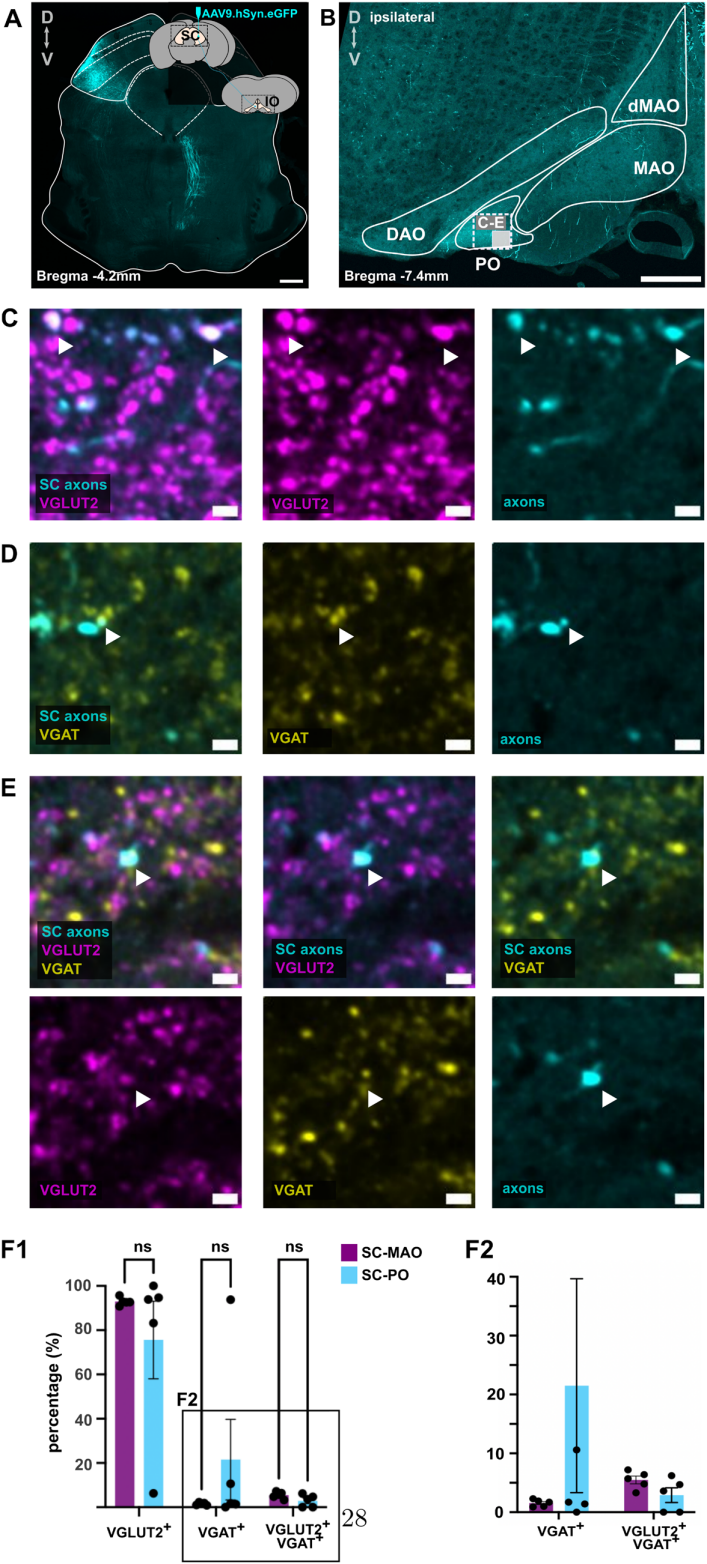
VGLUT2 and VGAT labeling in the principal olive. **A** Anterograde labeling of SC neurons using AAV9.hSyn.eGFP, showing labeled SC axons in the principal olive (PO), as depicted in **B**. The 100x100 $\mu$m square highlights region of interest (ROI) for 40x confocal imaging of labeled VGLUT2, VGAT, and SC axons. **C** A 40x confocal acquisition of a section of the PO, indicated by the square in panel B, showing labeled SC axons and VGLUT2. **D** Confocal acquisition of the same section of PO showing labeled SC axons and VGAT, and **E** panels showing SC-PO axons colabeled with both VGLUT2 and VGAT. The white triangles indicate some SC boutons that are collocalized with VGLUT2 and VGAT. **(F1)** Bar plot showing the percentage of SC axons that are VGLUT2+, VGAT+, VGLUT2+ and VGAT+ in MAO and PO. Dots represent data from five independent experiments. Multiple comparisons using two-way ANOVA is used to compare the percentage of VGLUT2+, VGAT+, and VGLUT2+/VGAT+ boutons in SC-MAO and SC-PO. **(F2)** The zoom in of VGAT+ and VGLUT2+/VGAT+ boutons. The scalebar of 200 $\mu$m for panel A,B and 5 $\mu$m for panel C-E

Although it has long been presumed that IO inputs originating outside of the cerebellar nuclei are primarily glutamatergic, recent findings have revealed additional complexities (Hoogstraten et al. 2024). We used immunohistochemistry to confirm the neurotransmitter identity of SC-MAO and SC-PO axons (Figs. 4, 5). SC axons were anterogradely labeled, followed by labeling of the vesicular glutamate transporter 2 (VGLUT2) and the vesicular $\gamma$-aminobutyric acid (GABA) transporter (VGAT) on SC-MAO (Fig. 4C-D) and SC-PO boutons (Fig. 5C-D). We then examined the colocalization of VGLUT2 and VGAT in the virus-labeled SC axonal boutons. As shown in Figs. 4E and 5E-F, we found that less than 10% of SC-IO boutons colocalized with both VGLUT2 and VGAT, and thus the glutamatergic neurotransmitter type is indeed the major form. In MAO, most of the SC axons were VGLUT2 + (average: 92. 94%), followed by VGLUT2+/VGAT+ (average: 5. 48%) and VGAT+ only (average: 1. 56%; Fig. 5F). The percentage of VGLUT2+ axons in MAO was higher than in PO, although this difference was not statistically significant ($P = 0.25$). In PO, most of the axons were also VGLUT2+ (75. 62%), followed by VGAT+ axons (21. 5%), which was higher than the percentage of VGAT+ in SC-MAO, though not statistically significant ($P = 0.18$). The percentage of VGLUT2+/VGAT+ axons in the PO was slightly lower than in the SC-MAO, at 2.9% ($P = 0.86$).

### Superior collicular axons target the IO neurons’ dendrites rather than soma

Locating the subcellular locations of SC axons on the IO neurons is informative regarding how their activation affects IO activity, given the dendritic gap junction coupling of IO neurons. Very few studies have examined subcellular axonal targeting patterns in IO (de Zeeuw et al. 1989), and the complex neuropil of IO, combined with the absence of layering, makes such investigations challenging.

**Fig. 6:**
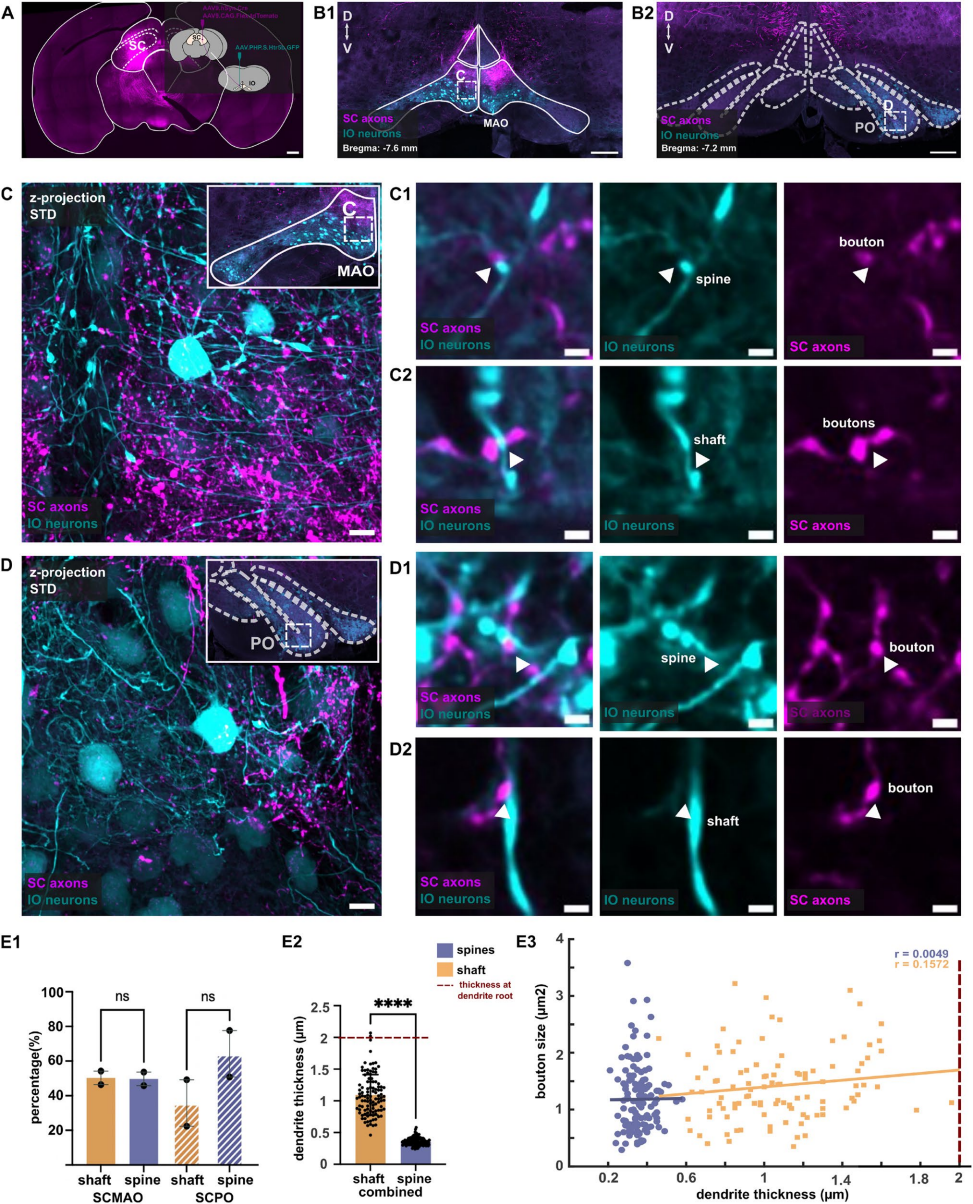
Identification of SC-IO synaptic contact along the SC dendrites. **A** viral tracer schematic to anterogradely label SC neurons using AAV9.hSyn.tdTomato and sparsely label the IO neurons using AAV.PHP.S.Htr5b.eGFP. **B** Inferior olive with tdTomato-labeled SC axons and eGFP-labeled IO neurons. Squares area in B indicates the ROI for 40X confocal imaging of the axons and IO neurons in MAO (**C)** and PO (**D)**. **(C1, D1)** shows synaptic contact found around the spines in MAO and PO while **(C2, D2)** shows synaptic contact found around dendritic shaft in both regions. **(E1**) Bar plot showing the number of synaptic contact on shaft and spines in SC-MAO and SC-PO. Multiple comparison ANOVA reveals that the higher number of spine contact in SC-PO is not statistically significant.**(E2)** Bar plot showing the dendrite thickness of synaptic contacts found on the shaft and spines. A Mann–Whitney test revealed that the differences in dendrite thickness between shaft and spine synapses is statistically significant. **(E3)** Scatterplot showing the relationship between dendrite thickness and bouton size for shaft and spine contacts, with the Pearson correlation indicating a weak correlation between bouton size and dendrite thickness

To examine the subcellular location of SC-IO synapses, we used our recently-developed IO-specific virus that drives sparse labeling of IO neurons (AAV.PHP.S.Htr5b.eGFP, Dorgans et al. (2022)), in combination with SC axons anterogradely labeled as described above (Fig. 6A-B). 40X confocal images were acquired from the MAO (Fig. 6C) and the PO (Fig. 6D) regions to analyze putative synaptic contacts. The labeled boutons were found in close proximity ($< 0.1\mu$m) to dendritic shaft and spines.

Of all boutons examined in SC-MAO (124 boutons, 2 animals), 50.3% were located on dendritic shafts, while in SC-PO, 64.2% of the boutons examined (123 boutons, 2 animals) were found apposed to the dendritic spines (Fig. 6E1). No significant differences were revealed in the distribution of axonal boutons apposed to shafts or spines in either SC-MAO and SC-PO ($P = 0.32$), and hence we combined the data for following analysis.

Next, to obtain insights on the position of the SC-IO boutons along the dendritic lengths, we examined the dendritic thickness at the synaptic contacts (Fig. 6E2). Intriguingly, we observed that the spine contacts resided on dendrites that were much thinner (average width: $0.36 \pm 0.005~\mu$m) than the dendrites at shaft contact points(average: $1.1 \pm 0.03~\mu$m). As the measured dendritic widths at the two categories of contact placements did not overlap, spine contacts predominantly occurred on the thinnest, putatively most distal dendrites.

**Fig. 7:**
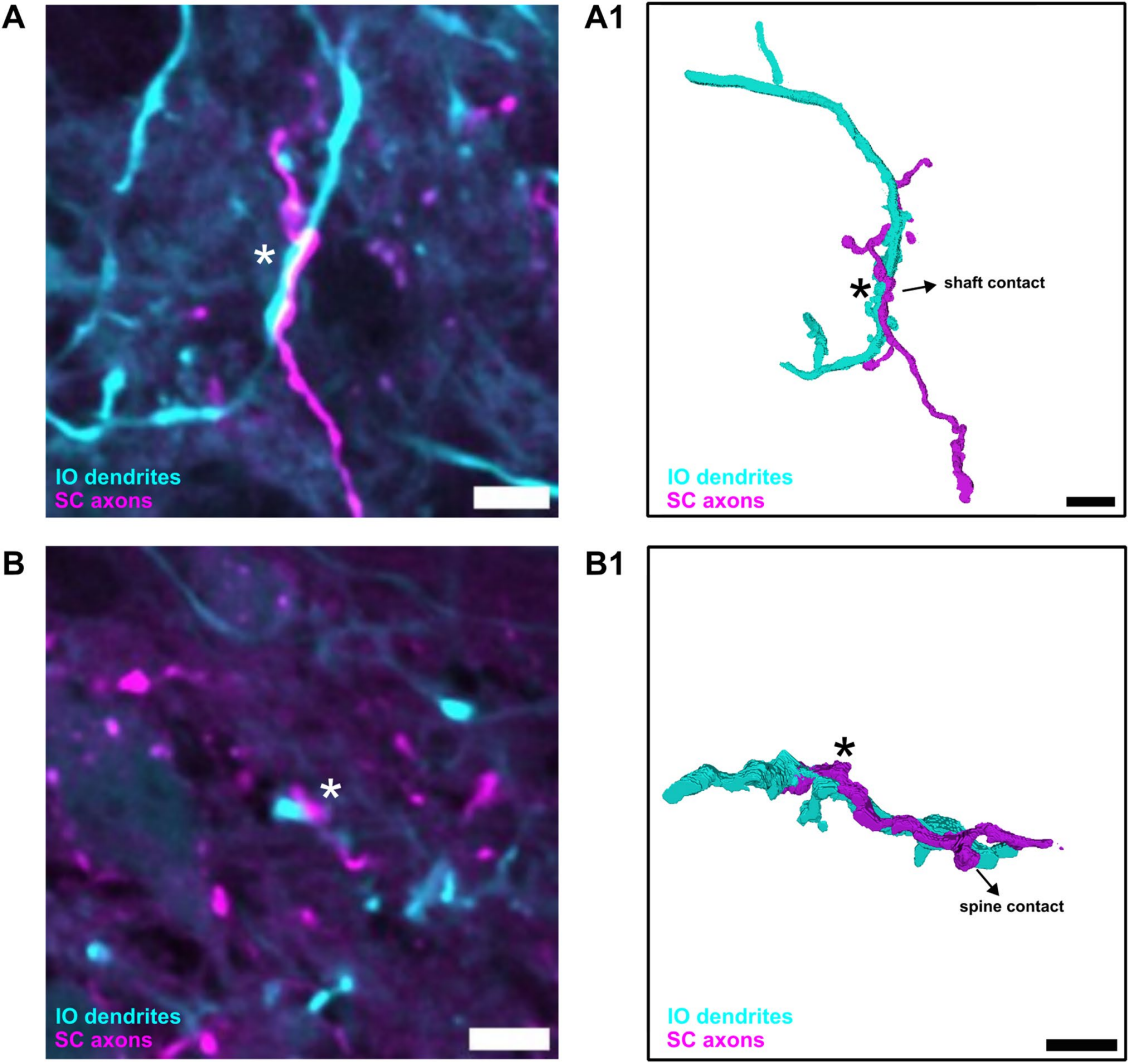
Reconstruction of SC-IO synapses. **A** Example confocal images of IO dendrites receiving two synaptic boutons from SC axon in caudal MAO and the PO dendrites (**B)**. **(A1, B1)** 2D visualization of 3D reconstruction of IO dendrites and SC axon from confocal image show in **A **and **B**. Scalebar 5 $\mu$m

Furthermore, we analyzed the correlation between dendritic thickness and the size of the presynaptic boutons at the dendritic shaft and spine. Using Pearson’s correlation, we found a weak positive correlation between bouton size and dendritic thickness for both types of synaptic contacts (Fig. 6E3). Furthermore, the scatterplot showed that the presence of dendritic spines decreases the likelihood of presynaptic boutons terminating on the shaft. Lastly, our confocal data also showed that an axon can have two or more boutons on the same IO dendrite, which we observed in both MAO and PO. The 3D reconstruction of this observation is shown in Fig. 7.

### In vivo effects of optogenetic activation of superior colliculus axons on the inferior olive activity

**Fig. 8:**
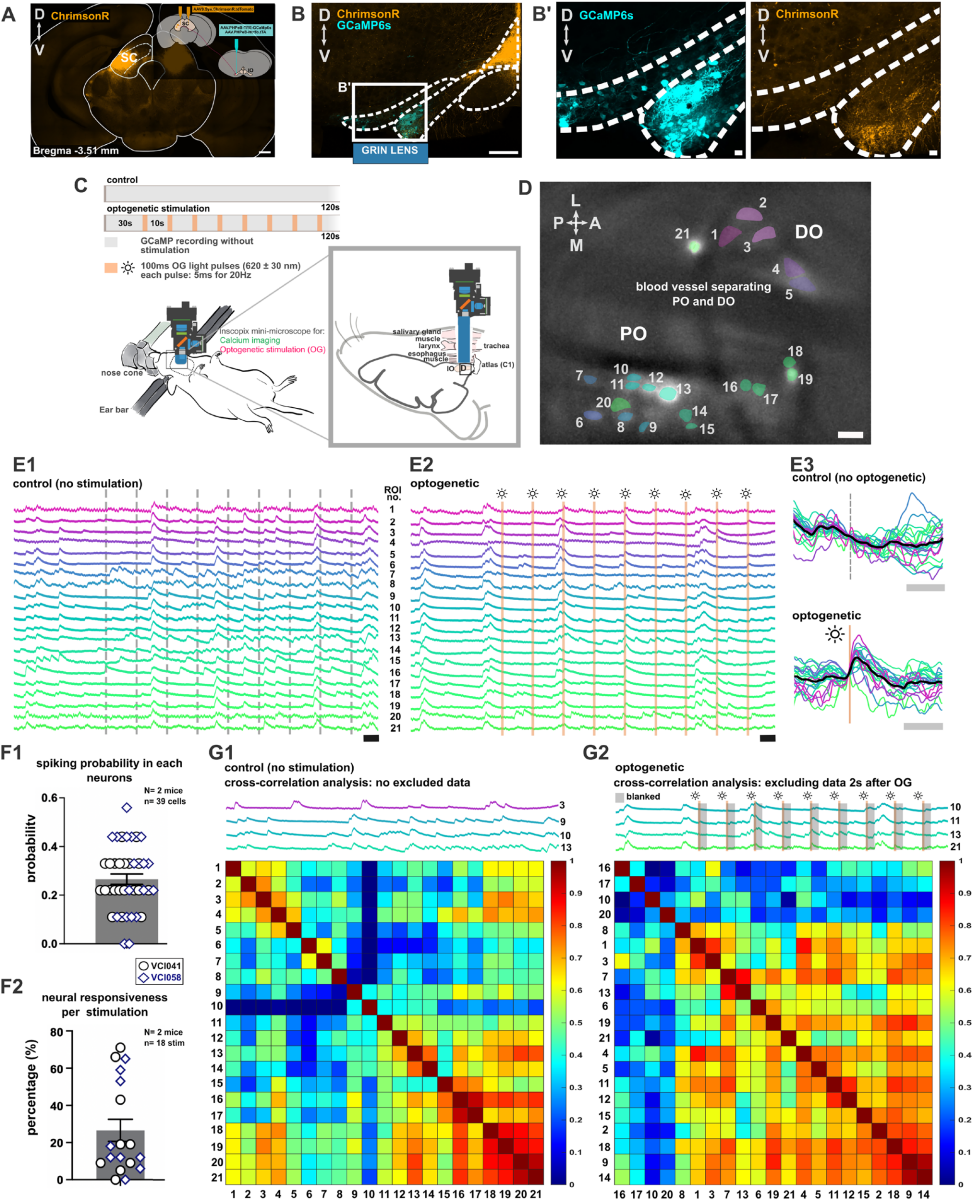
In-vivo calcium recording of IO neurons and the effect of optogenetic activation of SC axons on IO neurons activity. **A** Coronal section of midbrain containing SC neurons labeled with ChrimsonR coupled with tdTomato with a small schematic showing injection of AAV9.Syn.ChrimsonR.tdTomato into the SC and a mixture of AAV.PHPeB.TRE.GCaMP6s and AAV.PHPeB.Htr5b.tTA into the inferior olive. Scalebar 500 $\mu$m. **B** Coronal section of brainstem and the inferior olive with labeled SC axons and IO neurons. Scalebar 200 $\mu$m. **(B’)** Higher magnification of PO region highlighted in the square in **A**, showing the labeled SC axons and IO neurons. Scalebar 20 $\mu$m. **C** Schematic of ventral surgery to place the GRIN lens on the surface of ventral PO and recording settings for control (GCaMP6s recording only) and optogenetic trials. **(D)** Field of view of labeled and active IO neurons during recording with a scalebar of 20 $\mu$m. **(E1,E2)** Full traces of calcium recording during control and optogenetic trial. Each color corresponds to the colors of neurons in (**D**). Scalebar 5 s. **(E3)** Summary of fluorescence traces across stimulations, plotted before, during and after stimulation. In control trials, traces were aligned to the corresponding stimulation time points from optogenetic trials for comparison. Each color represents different neurons with average from all neurons indicated with black trace. Scalebar 2 s. **(F1)** Bar plot showing the probability of spiking in optogenetic trial. Each dot represents each neuron and different shapes represent different experiments. **(F2)** The percentage of responding neurons in each optogenetic stimulation with each dot representing each stimulation and different shapes representing different experiments. **(G1)** Cross-correlation matrix of recorded neurons during control and optogenetic trial. In optogenetic trial, the neuron activity during 2 s after each stimulation was trimmed before performing cross-correlation analysis

The preferred location of SC-IO boutons on distal dendrites raises the question of whether such synapses on distal dendrites are strong enough to evoke action potential in IO, or, would their function rather be related to efficiency of the gap junction coupling and network synchronization (Turecek et al. 2014). To investigate these questions, we examined neuronal activity in the soma using a novel surgical methodology that allows calcium imaging of GCaMP6s-expressing IO neurons and optogenetic activation of axons expressing optogenetic actuators in the ventral side of IO in living animals (Fig. 8 A-D, Guo et al. (2021); Guo and Uusisaari (2025)).

In these experiments, conducted in 2 animals, SC axons targeting the PO region were transfected with ChrimsonR and stimulated by brief pulses of light (10-second intervals; Fig. 8A-C). In response to light stimulation, the majority of recorded IO neurons generated spikes (Fig. 8E3), with an average response rate of one spike occurring in every three stimulations (Figure 8F1), similar to what has been reported previously for sensory stimulation of the IO (e.g. Lin et al. (2024)). On average, the response rate of the IO neighbourhood (putatively corresponding to an electrically-coupled cluster projecting to a single cerebellar microzone) at 26.6 ± 25.17 percent (Figure (8F2).

We further investigated whether stimulation affects the level of correlated activity within the IO. Examining the cross-correlation matrix between individual cells in trials without stimulation (Fig. 8 G1) showed that co-activation between neurons was high even without stimulation as expected (Guo and Uusisaari 2025). This co-activation and correlation increased even further in the trials with optogenetic stimulation (mean ± SEM for control trials: 0.3921 ± 0.024; for stimulation trials: 0.5817 ± 0.019; n = 39 cells; $P <0.0001$), even when the peri-stimulation periods were excluded from the construction of the cross-correlogram (Fig. 8 G2). Thus, the effect of excitatory input to the IO continues beyond immediate spike generation, possibly involving feedback activity across the olivocerebellar loop (Bengtsson et al. (2021)), and may affect generation of complex spikes for extended periods.

## Discussion

In this study, we describe in detail the projections between the mouse superior colliculus (SC) and the inferior olive (IO). In doing so, we provide evidence for a novel pathway linking the superior colliculus to a region of the principal IO that targets Crus 1 in the cerebellar cortex (Sugihara and Shinoda 2004). The SC axon terminals are largely glutamatergic and target dendritic shafts and spines in the distal regions of IO neurons. We also show that optogenetic activation of the SC terminals in the principal IO region can not only generate action potentials but also lead to enhanced correlation in activity of the olivary circuit even outside the stimulation period. Taken together, the observations suggest that the SC-IO projection is involved in motor coordination beyond basic posture and balancing. In the following, we will elaborate on the implications of findings and discuss future directions.

### Glutamatergic projection from the intermediate and deep layers of the rostro-lateral superior colliculus to the medial accessory and principal nuclei of the inferior olive

As the first step in examining subcellular targeting of superior colliculus (SC) axons, we anterogradely labeled SC neurons and confirmed previous findings showing projections from the medial and lateral parts of the SC to distinct parts of the contralateral cdMAO and weaker projections to the ipsilateral side. To our surprise, we found additional SC projections in the ipsilateral ventral principal olive (PO) (Fig. 1). This SC-PO projection was further validated through the retrograde labeling experiment (Fig. 2) using retrograde viral vector and CTB (Supplementary Figure 03). In the retrograde labeling experiment, SC neurons that project to the IO ("SC-IO" neurons) were observed exclusively in the intermediate and deep layers of the lateral SC, regardless of whether they target the MAO or PO ("SC-MAO" and "SC-PO" neurons, respectively). Similarly, no differences were found in the distribution of SC-MAO and SC-PO neurons along the rostro-caudal axis of SC, and the majority of all SC-IO neurons occupy the rostral end of SC (Fig. 2 D1-D2). Examination of SC neurons retrogradely labeled from either MAO or PO are very similar in size (average SC-MAO soma cross-section area: 193.7 ± 4.20 $\mu$m^2^; SC-PO: 203.4 ± 7.25 $\mu$m^2^, Fig. 3). Very few SC neurons were found to be double-labeled, indicating that divergence of projection into both IO regions is rare. When comparing the SC-IO neurons with other SC neurons in the same neighbourhood, it appears that the SC-IO neurons are slightly larger on average; however, as the efficacy of retrograde labeling is uncertain, we can not exclude the possibility that the "non-SC-IO"-population contains small SC-IO neurons that were weakly labeled. Finally, examination of the distribution of the presynaptic VGLUT2+ and VGAT+ boutons revealed no significant differences, and the majority of boutons were VGLUT2+ in both the SC-MAO and SC-PO neurons (Fig. 4,5).

### Superior colliculus axons target the distal dendrites of IO neurons

Since SC-IO boutons are predominantly glutamatergic, as indicated by the large proportion of VGLUT2+ SC boutons, input from the SC may evoke IO spikes. However, if the presynaptic boutons are located primarily on the distal dendritic spines, it is questionable to what extent activation of SC axons can evoke spikes in PO, or whether it would play mainly a modulatory role, possibly related to influences on effective electrical coupling via gap junctions residing on dendritic spines De Zeeuw et al. (1998); Devor and Yarom (2002); De Gruijl et al. (2014); Leznik and Llinás (2005); Lefler et al. (2014). Therefore, we examined the subcellular localization of SC-IO synapses and investigated if there are differences between SC-MAO and SC-PO.

We identified SC boutons residing in close proximity ($< 0.1\,\mu$m) or apposed to IO neurons by sparse viral labeling. Notably, we only found boutons on dendrites and were unable to confirm the presence of somatic contacts. Along the dendrites, we found putative contacts both on the dendritic shafts and spines. There was no evident preference for shaft over spine contact, but dendritic thickness analysis showed that shaft and spine contacts are found in different parts of the dendrites. Shaft contacts were found on thicker dendrites, presumably closer to the soma, whereas contacts on spines were found in regions where the dendrites are thinnest. Although we can not yet propose functional significance for this observation, the selectivity is intriguing as spines do exist along more proximal dendrites (see Fig. 7). We also note that an axon can have multiple synaptic boutons contacting the same location on a dendrite, which can amplify the synaptic input strength.

### Optogenetic activation of superior colliculus axons drives calcium events in inferior olive neuronal population in vivo

The presence of glutamatergic synaptic contacts at the dendritic spines might allow at least two distinct effects of activity in the SC-IO pathway: (i) SC activation increases the probability of spiking in IO neurons and/or (ii) SC activation influences overall co-activation of the IO network. To test these possibilities, we optogenetically activated SC axons and examined the effect on the calcium activity of the IO in anesthetized mice.

Our findings demonstrated that activation of the SC-PO axons not only can evoke postsynaptic spikes but also increase synchrony within the IO population. Full trial traces in figure 8E-F1 show that optogenetic activation of the SC boutons evokes spikes in approximately 1 out of every 3 stimulation events, despite the contacts seemingly residing distal to the soma, demonstrating efficiency comparable to strong sensory inputs, such as airpuff (as evidenced by triggering complex spikes in cerebellar Purkinje neurons, Ju et al. (2019); Guo and Uusisaari (2025)). This strength may be attributed to the local branching of the axons and multiple synapses on single neurons which could facilitate spike generation in IO. This finding focusing on the PO complements previous studies showing that SC activation induces complex spikes in the vermis, which receives climbing fiber projections from the MAO (Akaike 1985.

A previous study showed that glutamatergic input near gap junctions can modulate electrical coupling between neurons (Turecek et al. 2014). Here, we show that stimulation of the SC-PO axons increases correlated activity among the IO neurons (Fig. 8 G2). This finding aligns with our anatomical observation of SC-IO synapses on dendritic spines, where the gap junctions that may underlie the correlated activity are located.

### Methodological considerations

The reliability of any neuronal tracing study is fundamentally limited by the accuracy of the methodology, and ours is not an exception. Notably, despite our injection protocol being highly reproducible and accurate, diffusion of the viral injectate is a parameter that is difficult to control. Hence, it is necessary to consider potential caveats in our description of the novel SC-PO pathway.

The SC is a relatively large structure that is easily targeted from dorsal side of the brain, but spill-over of the viral particles beyond the borders of the SC is still possible. However, as shown in Supplementary Figure 02, no labeled neurons were found in the neighbourhood of the SC when a retrograde virus was injected into the IO, suggesting that no prominent projections from these regions exist.

Precise targeting of the IO subnuclei is more challenging but feasible, as demonstrated by the absence of labeling in the region immediately bordering the IO. Notably, tracer injection into the principal IO systematically labels large neurons in the medullary reticular region (seen in Figure 2 A3, A6 and B2), in line with the known reticulo-olivary projection (Bishop 1984; Bishop and King 1986).

Another confounding factor in our work relates to the comparison of soma sizes between IO-projecting and non-IO-projecting SC neurons (Fig. 3). As the efficacy of retrograde viral labeling is unknown, it is possible that the population classified as "non-IO SC neurons" includes cells that in fact do project to the IO but did not get strongly labeled. Thus, while the apparent difference in soma sizes is suggestive of a morphological difference between IO-projecting and "other" SC cells, ultimately, such a conclusion will require additional examination by means of, e.g. molecular markers. Additionally, because it is challenging to ensure that a viral injection into the MAO region does not spread across the midline and potentially reach the contralateral MAO, and given the known bilateral projections from the SC to the MAO, we did not compare the density of labeled SC-MAO neurons between the contralateral and ipsilateral sides.

Finally, while confirming the accuracy of the labeling of the SC-IO axons with ChrimsonR in the live imaging experiments, we observed the presence of labeled SC axons in the more dorsal region of the medial accessory olive (encompassing the beta subnucleus, the cap of Kooy, and the ventrolateral protrusion). We suspect that this labeling may result from the diffusion of anterograde tracers into adjacent regions, such as the periaqueductal gray (PAG) or inferior colliculus (IC). However, as the labeling in the ventral PO closely matched the results presented with more restricted injections and the extraneously labeled axons in dorsal MAO were far (more than 500 um) from the optical stimulation location, we consider it reasonably safe to assume that the optogenetic activation is limited to SC axons.

### Functional considerations

The anatomical features and functional properties of the SC-IO pathway can provide insights into its potential behavioral relevance. The SC is well known for its role in mediating orienting-related movements (Masullo et al. 2019; Zhou et al. 2023; Solié et al. 2022), and the SC-MAO pathway supports this function. MAO targets Purkinje neurons in the cerebellar vermis, involved in postural control and balancing that is essential for orienting movements. However, the SC-PO pathway appears to contribute to a different aspect of movement orientation. PO neurons target Purkinje cells in Crus I, which are associated with orofacial and forelimb movements (Gaffield et al. 2022; Bryant et al. 2010; Saint-Cyr 1983). Thus, presence of a SC projection to the PO in mice suggests the potential involvement in orienting behaviors that extend beyond the previously-recognized roles in head (Masullo et al. 2019) and eye (Lee et al. 2007) movements. Observation of the location of SC-IO neurons in rostro-lateral SC offers important insights into the nature of information relayed by this pathway. In primates, activation of the rostral intermediate SC is associated with the control of gaze stabilization (Marino et al. 2023) and the generation of microsaccades during visual fixation (Hafed et al. 2009). In fact, suppressing activity in the rostral half of the deep SC in monkeys results in a biased perception of target position during visual fixation (Goffart et al. 2012). A similar functional specialization may exist in rodents; however, it is more difficult to assess due to their lack of foveal vision and the resulting differences in visual fixation behavior.

Regarding the function of lateral parts of the SC, a recent study showed that manipulation of glutamatergic neurons in the lateral SC can affect the precision of forelimb movement in mice (Chaterji et al. 2025). Another study conducted in macaques demonstrated that microstimulation of lateral SC neurons can induce arm movement (Philipp and Hoffmann 2014). Given that SC-IO neurons reside in the lateral SC, which processes signals from the lower visual field (Cang et al. 2018; Ito and Feldheim 2018), the SC-PO pathway might play a role in hunting- or prey-grasping-related behaviors involving orofacial or forelimb movement.

## Conclusion and future directions

Our study provides a mesoscale and microscale description of the SC-IO pathway, including a novel SC projection to the PO. We showed that the pathway is mainly glutamatergic with putative synapses found on the shafts of the thick dendrites and on the spines of the distal and most thin dendrites. We further showed that optogenetic activation of the SC axons in the IO can evoke spikes and increase the synchrony among the IO neuronal population. The results suggest that the influence of SC on cerebellar processing can be more extensive than previously considered. Although the SC-MAO pathway is known to play a role in postural control and balance, function of the SC-PO pathway may be rather different. Its projection to Crus I -related subregion of the IO suggests potential involvement in orofacial and forelimb movement or even non-motor functions. Given that most SC neurons involved in this pathway are located in the lateral SC, representing the lower visual field, an exciting possibility is that the SC-PO pathway may play a role in forelimb control in hunting-related behaviors (Chaterji et al. 2025). Future experiments comparing the effects of SC-PO and SC-MAO activation or inhibition will be important to understand the role of the olivocerebellar system in orienting related movement.

## Methods

### Animals

In this study, wild-type C57BL/6J male mice (12–16 weeks old; CLEA Japan, Shizuoka, Japan). Animals were housed in rich-environment cages with extra bedding, a tunnel and a running wheel. Experiments were conducted in strict adherence to the approved guidelines by the Okinawa Institute of Science and Technology (OIST) and the Institutional Animal Care and Use Committee (IACUC), within a facility accredited by the Association for Assessment and Accreditation of Laboratory Animal Care (AAALAC International). 4 mice were used for anterograde labeling of SC neurons, 3 mice were used for retrograde labeling of IO afferents, 3 mice were used to label SC axons and sparsely label IO neurons, and another 3 mice were used for anterograde labeling of SC neurons combined with immunofluorescece. 2 mice were used for in-vivo calcium imaging experiment.

### Surgical procedures for viral expression

Mice were anesthetized with 5 % isofluorane (delivered by means of the SomnoSuite vaporizer system, Kent Scientific, CT, USA) and placed in the stereotaxic frame (KOPF instruments, CA, USA) equipped with a heat pad (38$^{\circ }$C; TMP-5b, Supertech Instruments). During surgery, the animal was kept under anesthesia with 1.5–2.5% isofluorane delivered through a nose cone. Precise alignment of the head and body is crucial for accurate targeting of deep structures such as the IO (Dorgans et al. 2022; Guo et al. 2021; Guo and Uusisaari 2025). The scalp was shaved and local anesthesia was administered using Xylocaine gel (Xylogel, gel 2%, Aspen, Japan), and the skin was disinfected and incised with a scissor to expose the skull. Subsequently, small craniotomies (approximately 1 mm in diameter) were opened using a handheld drill (Surgic XT Plus drill, NSK Dental, Japan).

Following the placement of viral mixtures into the quartz capillary pipette, the pipette was slowly (0.2 mm/s) maneuvered into the desired location within the brain tissue. Using bregma as a reference point, the coordinates of anterio-posterior (AP): 3.3mm, dorso-ventral (DV): 2.1mm, and medio-lateral (ML): 0.7mm and 1.2mm for targeting medial and lateral regions of the superior colliculus. For the medial inferior olive: AP: 6.1mm, ML: 0.4mm, DV: 6.72mm and for the principal olive: AP: 6.4mm, ML: 0.2mm, DV: 6.72mm. At these locations, 20–40 nl of viral mixtures were injected with a speed of 20 nl/min. Approximately 5–10 min after the completion of the viral delivery, the pipette was retrieved from the tissue with slow speed. The skin was cleaned and the incision was sutured, followed by subcutaneous administration of 5 mg/kg of Rymadil (Zoetis, NJ, USA) to minimize the post-operative discomfort. Following stereotaxic viral injections, mice were placed on a heating pad and monitored continuously until they regained full mobility. Mice were returned to their cage with a familiar cage mate to reduce stress. Health status, grooming, and overall activity were monitored daily for at least 3 days post-surgery. Animals showing signs of distress, infection, or abnormal behavior were promptly evaluated and treated in accordance with institutional animal care protocols.

### Viral vectors

For anterograde labeling of superior colliculus neurons (SC), we use AAV9.hSyn.eGFP (final titer: $7 \times 10^{11}$ vg/ml, 50465, Addgene) and a mixture of AAV9.hSyn.Cre (105553, Addgene) or AAV9.CamKII.Cre (105558, Addgene) with AAV9.CAG.Flex.tdTomato (28306, Addgene). These viral vectors were carefully mixed and subsequently diluted in phosphate buffered saline (PBS) to achieve a final titer of $1.7 \times 10^{12}$ vg/ml.

To retrogradely label the afferents in different inferior olive subnuclei (IO), we used AAVrg.CAG.tdTomato (final titer: $3.5 \times 10^{12}$ vg/mL, 59462, Addgene) and AAVrg.CAG.eGFP (final titer: $3.5 \times 10^{12}$ vg/mL, 37825, Addgene) in the medial accessory olive (MAO) and principal olive (PO).

The AAV9.hSyn.Cre and AAV9.CAG.Flex.tdTomato mixture, along with AAV.PHP.S.Htr5b.eGFP (final titer: $5 \times 10^{12}$ vg/mL, OIST), was used to label SC axons and sparsely label IO neurons to identify the location of SC synapses along the IO. For *in vivo* calcium imaging of IO neurons, we used a mixture of IO-specific viruses: AAV.PHP.eB.TRE.GCaMP6s (final titer: $2 \times 10^{11}$ vg/mL, OIST) and AAV.PHP.eB.Htr5b.tTA (final titer: $2 \times 10^{10}$ vg/mL, OIST Dorgans et al. (2022)). Furthermore, AAV9.Syn.ChrimsonR.tdTomato was used for optogenetic activation of SC axons.

### Tissue processing and immunohistochemistry

Two to three weeks after injection of viral tracers into the superior colliculus (SC), the animals were transcardically perfused with PBS followed by 4% PFA (w/v, in PBS). The brains were extracted and postfixed in 4% PFA solution for 2–3 h at room temperature, after which they were stored in PBS at 4 $^{\circ }$ C until sectioning. The brains were sectioned using a vibratome (Model 5100MZ-plus, Campden Instruments, Loughborough, UK) equipped with ceramic blades (Model 7550–1-C, 38 x 7 x 0.5 mm, Campden Instruments, Loughborough, UK). The slices were mounted with Vectashield (Vector laboratories, CA, USA) or Prolong Glass (Invitrogen, MA, USA) and #1.5 coverslip glass (Harvard Apparatus, MA). For immunohistochemistry, the brains were sectioned either with with the vibratome or with a cryostat (CM1950, Leica, IL, USA) equipped with blades (Disposable Blades DB80LX, Leica, IL, USA). The brains that were sectioned with the cryostat were cryoprotected by submerging them in a 10% sucrose solution (w/v, in PBS) for several hours at room temperature, followed by being stored overnight in a 30% sucrose solution (w/v, in PBS) before sectioning. To verify injection sites in the SC, the rostral parts of the brains were sectioned coronally at 50 $\mu$m or 100 $\mu$m. These sections were washed 2x in PBS for 10 min at room temperature, followed by 3 X 5 min washes in 0.1M phosphate buffer (PB) at room temperature, after which they were mounted on an objective glass with Prolong glass mounting medium (Invitrogen, MA, USA) and #1.5 coverslip glass (Harvard Apparatus, MA, USA).

The brainstems were sectioned separately at 50 $\mu$m in coronal orientation, also using the vibratome with ceramic blades or cryostat. For immunostaining, every second brainstem section was stained freely-floating using anti-VGluT2 (Polyclonal, Thermo Fisher, MA, USA) and anti-VGAT (Polyclonal, Alomone labs, Jerusalem, Israel). The sections were first washed 4 times in PBS at room temperature for at least 10 min. Subsequently, they were blocked and permeabilized in PBS containing 10% Normal Goat Serum (NGS, Abcam, Cambridge, UK) and 0.5% Triton X (Thermo Fisher, MA, USA) for 2 h on a shaker (100 rpm), followed by primary antibody incubation. For this step, the slices were incubated for 48 to 72 h at 4$^{\circ }$C in a solution of PBS with 2% NGS and 0.1% Triton X and anti-VGluT2 (1:500) and anti-VGAT (1:2000) primary antibodies. The sections were subsequently washed 5 times for 10 min in PBS at room temperature, before secondary antibody incubation for 2 h at room temperature, in a PBS solution with 2% NGS, 0. 1% Triton X and secondary antibodies. The secondary antibodies used were goat-anti rabbit-A647 (Invitrogen, MA, USA) and goat-anti guinea pig-A555 (Invitrogen, MA, USA), both at a concentration of 1:2000. Finally, sections were washed 2 times for 10 min in PBS, followed by 3 washes in 0.1M phosphate buffer (PB), before being mounted using the same procedure as viral-labeled sections.

### Confocal image acquisition and image processing

Z-stack images of sections labeled using viral and immunohistochemical methods were acquired using a Zeiss LSM 880 confocal system (Carl Zeiss Microscopy, Jena, Germany). For overview scanning of the entire cortex and brainstem, a 10x objective (Plan-Apochromat 10x, NA = 0.5; Carl Zeiss Microscopy, Jena, Germany) with a 6 $\mu$m z-step was used. To scan the inferior olive (IO), a 20xobjective (Plan-Apochromat 20x, NA = 0.8; Carl Zeiss Microscopy, Jena, Germany) with a 3–6 $\mu$m z-step was used. For the superior colliculus (SC), a 10x objective (Plan-Apochromat 10x, NA = 0.5; Carl Zeiss Microscopy, Jena, Germany) with a 6 $\mu$m z-step was used. Higher-magnification images were acquired using a 40x oil-immersion objective (Plan-Apochromat 40x Oil DIC M27, NA = 1.4; Zeiss Immersol oil; Carl Zeiss Microscopy, Jena, Germany) with 0.07$-$0.1 $\mu$m z-steps, adjusted to match the x and y resolution. All images were acquired at 1024x1024 pixel resolution.

For multichannel imaging, the following excitation/emission settings were used: 488 nm/490–535 nm for eGFP, 561 nm/470–655 nm for tdTomato, and 633 nm/638–758 nm for the infrared channel. Acquisition parameters were optimized for each experiment but kept constant across samples within the same experimental group. After acquisition, z-stacks were processed using a 3D Gaussian filter with $\sigma$ = 1 pixel in x, y, and z dimensions, and converted into 2D images using a standard deviation projection along the z-axis.

### Percentage of SC-IO axons labeling across IO subnuclei

After acquiring images from the IO, we classified them into three rostro-caudal regions: caudal IO, middle IO, and rostral IO. The subnuclei within each IO region were manually delineated using Fiji (Image J, DC, USA). We classified the subnucleus a, b and c of the inferior olive (IOA, IOB, and IOC) as ventral MAO and classified IOVL (inferior olive, ventrolateral protrusion), IOBe (inferior olive, beta subnucleus), IOK (inferior olive, cap of Kooy), and IODM (inferior olive, dorsomedial) as dorsal MAO.

For each rostro-caudal IO region, we quantified the intensity of labeled SC axons in different IO subnuclei using slices from five animals. Intensity values were normalized to the maximum intensity value for each animal (see Supplementary Table 1) to generate the values presented in Fig. 1H1. Normalized values below 0.2 were considered indicative of passing axons or background noise from image acquisition.

### Soma areas and position of the SC-IO neurons

To locate the labeled SC-IO neurons along the rostro-caudal axis, the midline position of the acquired confocal images of the midbrain was manually aligned and the coordinates of each slice were matched to the bregma position using the Mouse Brain Atlas (Paxinos and Franklin 2001). The centers of mass of the soma boundaries were used as x-y positions, and their distance to the midline and SC surface was calculated. To further map the labeled neurons along the mediolateral axis (M-L), the locations of the soma were transformed into ’angular space’, following the conventions described in Benavidez et al. (2021).

### Neuronal tracing and identification of the SC-IO putative synaptic contact

To identify the location of synapses, processed confocal images of IO neurons and SC axons were imported into Vaa3D (Peng et al. 2014), an open-source platform for high-resolution 3D visualization and neuron tracing. IO neurons and SC axons were manually traced in three dimensions, and points of close apposition were identified as putative synapses. The number of synapses was quantified and exported to Excel and each contact was manually classified as either spine- or shaft-contact. The thickness of IO dendrites at the sites of putative SC-MAO and SC-PO synapses was manually measured.

Next, to quantify the ratio of VGluT2+ to VGAT+ boutons in SC fibers, we identified several 100 x 100 $\mu$m regions of interest within the caudal medial accessory olive (cdMAO) and the principal olive (PO) eGFP+ SC axons in immunolabeled slices. We selected ROIs per animal (two ROIs within the MAO and one ROI in the ventral PO, on both sides).

Z-stacks of these ROIs were acquired using a confocal microscope with a 40x objective. The images are further divided into four subregions to facilitate analysis. In each of the subregions, we applied noise reduction using the 3D Gaussian filter ($\sigma$ x,y,z =1 pixel) and manually quantified the percentage of GFP+ boutons labeled with VGluT2, VGAT, both and none, using the following procedures in the FIJI software. Afterwards, the analysis was performed in two steps using custom FIJI macros.

The first step aimed to identify eGFP+ boutons (indicating that the origin of the axon was in the SC). For the PO, the number of boutons was rather low, allowing us to identify and label all boutons in the image. This was done by manually drawing ROIs in FIJI using the free-hand or polygon selection tool. For densely labeled boutons in MAO it was not feasible to label all boutons individually. Instead, a random subset of boutons was selected. This selection process was performed manually with the assistance of a TANGO macro (Ollion et al. 2013). The macro identified local maxima that exceeded an intensity threshold of 2500 using a local maximum filter from the ImageJ 3DSuite package. The macro then randomly presented to the person analyzing (observer) with one of the identified local maxima, allowing them to draw an ROI if the local maximum was deemed to correspond to a bouton. This process continued until all local maxima in the image were evaluated or a predetermined threshold (set at 50 boutons) was reached for the subregion.

The second step aimed to determine whether the boutons identified in step one were labeled with VGluT2 or VGAT. A macro presented each ROI to the user for manual categorization as labeled, unlabeled, or doubtful for both the VGluT2 and VGAT channels. For analysis purposes, boutons marked as "doubtful" were treated as unlabeled. Furthermore, users could exclude any bouton from the analysis if they were not completely confident that ROI represented a synaptic bouton. To avoid subjective bias, each step was analyzed by different observers. For example, if step one was conducted by observer A, step two was performed by observer B.

### In-vivo calcium imaging of IO neuronal population and pptogenetic activation of SC axons

For calcium imaging experiments, we used a ventral access surgery. The procedure is conducted under deep anesthesia (isofluorane 1.5–2%) and the animal is not allowed to regain consciousness after the recording is completed.

Two to three weeks after injecting AAV9.Syn.ChrimsonR.tdTomato into the superior colliculus (SC) and AAV.PHPeB.TRE.GCaMP6s along with AAV.PHPeB.Htr5b.tTA into the inferior olive (IO), mice were anesthetized with 5% isoflurane for ventral surgery to allow placement of the GRIN lens (Guo et al. 2021). Prior to surgery, the thigh and throat were shaved, with thigh sensors used to monitor oxygen and breathing rate, and a rectal probe used to monitor body temperature.

An incision was made on the throat after the application of lidocaine. The salivary glands were displaced and the sternothyroid muscles overlying the trachea were removed for tracheotomy. The thyroid gland was removed and a thread was secured around the tracheal ring to stabilize it before connecting it to a pre-made intubation tube, ensuring the maintenance of the anesthesia. The esophagus, larynx, and surrounding tissues were removed, exposing the occipital bones, ventral arch, and anterior tubercle of the atlas. The ventral arches of the atlas and anterior tubercle were removed using a rongeur, and the surrounding occipital bone was cut to enlarge the field of view. The cartilage overlying the dura was removed to expose the ventral IO to have a clear view during calcium recording.

A GRIN lens (9 mm length, 1 mm diameter) connected to an Inscopix mini-microscope was placed on the ventral IO surface. For the GCaMP6s recording, blue LED light (455 ± 8 nm) was used, while red light (620 ± 30 nm) was used for optogenetic stimulation. The 40 Hz recordings lasted for 120 s each. For optogenetic stimulation, a 100-ms pulse train (20 Hz, 5 ms pulses) was delivered 30 s after the recording began, with nine repetitions with a 10-second interval between the stimulations. The recordings were spatially filtered and motion corrected using Inscopix Data Processing (IDP) software. The regions of interest (ROIs) were manually traced, and the fluorescence traces were analyzed and visualized in MATLAB.

### Data analysis and statistics

For the analysis of SC-IO neuron coordinates and soma size, all detectable SC-PO and SC-MAO neurons were manually delineated in Fiji following image processing, and saved as Regions of Interest (ROIs). Neurons with poorly defined soma boundaries or very faint fluorescence were excluded. Each ROI was classified as SC-MAO, SC-PO, or co-labeled (both), and soma area was subsequently measured. Measurements were imported into GraphPad Prism for data visualization and statistical analysis. Values are reported as mean ± SEM.

To compare cumulative distributions of SC-IO, non–SC-IO, SC-MAO, and SC-PO neurons, plots were generated using custom MATLAB code, and statistical comparisons were performed using the Kolmogorov–Smirnov test. The spatial distributions of SC-MAO and SC-PO neurons along the rostro-caudal and medio-lateral axes were also visualized using custom-written MATLAB scripts.

IHC analysis was assisted by a custom FIJI macro adapted from a previously published tool (Ollion et al. 2013). Results were recorded and summarized in a Microsoft Excel file, and data visualization was performed in GraphPad Prism. For synapse localization analysis, GraphPad Prism was used to visualize the distribution of synapse positions and comparison of the spine vs shaft contact.

Following spatial filtering and motion correction, all subsequent analysis and visualization of the calcium imaging data were performed using custom-written MATLAB code. Fluorescence traces were low-pass filtered and aligned to stimulus onset for visualization. Cross-correlation matrices were generated in MATLAB, along with the mean cross-correlation value between trials.

## Supplementary Information

Below is the link to the electronic supplementary material.

Supplementary file 1 (pdf 25 KB)

Supplementary file 2 (PNG 6917 KB)

Supplementary file 3 (PNG 4755 KB)

Supplementary file 4 (PNG 3368 KB)

## Data Availability

All data supporting the findings of this study are available within the paper and its Supplementary Information.
