# Supplementary material for: Anatomical and functional examination of superior colliculus projections to the inferior olivary in mice
Source: Brain Struct Funct. 2025 Dec 8;231(1):2. doi: 10.1007/s00429-025-03032-1 (PMC12686038; doi:10.1007/s00429-025-03032-1)
Supplement: Supplementary file 1 — (pdf 25 KB) [file 429_2025_3032_MOESM1_ESM.pdf]

Table 1: SC axons intenstity across IO subnuclei

(normalized to max intensity value in each sample)

		MAO_contra	MAO_ipsi	dorsalMAO_	dorsalMAO_i	IOPr_contra	IOPr_ipsi	IOD_contra	IOD_ipsi
**caudal IO**	**SC080**	1	0.1	0.7	0.1				
		1	0	0.3	0				
		1	0.1	0.7	0.1				
		1	0	0.3	0				
		1	0.1	0.7	0.1				
		0.8	0	0.2	0				
	**SC081**	0.5	0	0.2	0				
		0.5	0.1	0.3	0.1				
		1	0.1	0.4	0.1				
		1	0.1	0.6	0.1				
		0.9	0.1	0.3	0.1				
		0.9	0.1	0.5	0.1				
	**SC010**	0.3	0	0.2	0				
		1	0.1	0.3	0				
	**SC011**	1	0	0.3	0				
		0.9	0.1	0.2	0				
		1	0	0.3	0				
	**SC014**	0.9	0	0.3	0				
		0.9	0.1	0.4	0.1				
**middle IO**	**SC080**	0.9	0.8	0.19	0.18	0.23	0.12	0.51	0.51
		0.42	0.2	0.05	0.05	0.05	0.03	0.14	0.14
		0.97	0.98	0.33	0.26	0.12	0.11	0.48	0.48
		0.27	0.23	0.1	0.07	0.02	0.02	0.11	0.11
	**SC081**	0.25	0.19	0.05	0.04	0.02	0.02	0.12	0.12
		0.34	0.31	0.07	0.05	0.04	0.04	0.19	0.19
	**SCIHC010**	0.76	0.3	0.07	0.06	0.03	0.02	0.13	0.13
		0.66	0.42	0.12	0.08	0.06	0.06	0.27	0.27
	**SCIHC011**	0.35	0.17	0.03	0.03	0.03	0.02	0.13	0.13
		0.17	0.17	0.06	0.04	0.04	0.04	0.1	0.1
	**SCIHC014**	0.31	0.23	0.03	0.03	0.02	0.03	0.1	0.1
		0.15	0.18	0.07	0.07	0.05	0.05	0.12	0.12
**rostral IO**	**SC080**	0.18	0.19	0.23	0.17	0.52	0.61	0.35	0.35
		0.04	0.04	0.05	0.04	0.11	0.14	0.08	0.08
		0.27	0.26	0.22	0.2	0.71	0.78	0.49	0.49
		0.06	0.06	0.05	0.05	0.15	0.18	0.1	0.1
	**SC081**	0.17	0.19	0.13	0.1	0.3	0.45	0.25	0.25
		0.3	0.34	0.22	0.16	0.55	0.52	0.45	0.45
		0.1	0.09	0.08	0.07	0.19	0.31	0.18	0.18
		0.18	0.15	0.13	0.11	0.33	0.37	0.32	0.32
		0.05	0.05	0.05	0.04	0.15	0.33	0.1	0.1
		0.09	0.09	0.09	0.07	0.26	0.29	0.19	0.19
	**SCIHC010**	0.11	0.13	0.09	0.09	0.28	0.33	0.18	0.18
		0.06	0.07	0.06	0.05	0.13	0.13	0.14	0.14
		0.07	0.08	0.06	0.07	0.22	0.2	0.16	0.16
	**SCIHC011**	0.06	0.06	0.05	0.05	0.15	0.15	0.13	0.13
		0.06	0.06	0.06	0.05	0.16	0.15	0.14	0.14
	**SCIHC014**	0.05	0.06	0.06	0.04	0.2	0.13	0.12	0.12
		0.06	0.06	0.03	0.03	0.15	0.21	0.09	0.09
