# Supplementary figures and images for: Anatomical and functional examination of superior colliculus projections to the inferior olivary in mice
Source: Brain Struct Funct. 2025 Dec 8;231(1):2. doi: 10.1007/s00429-025-03032-1 (PMC12686038; doi:10.1007/s00429-025-03032-1)

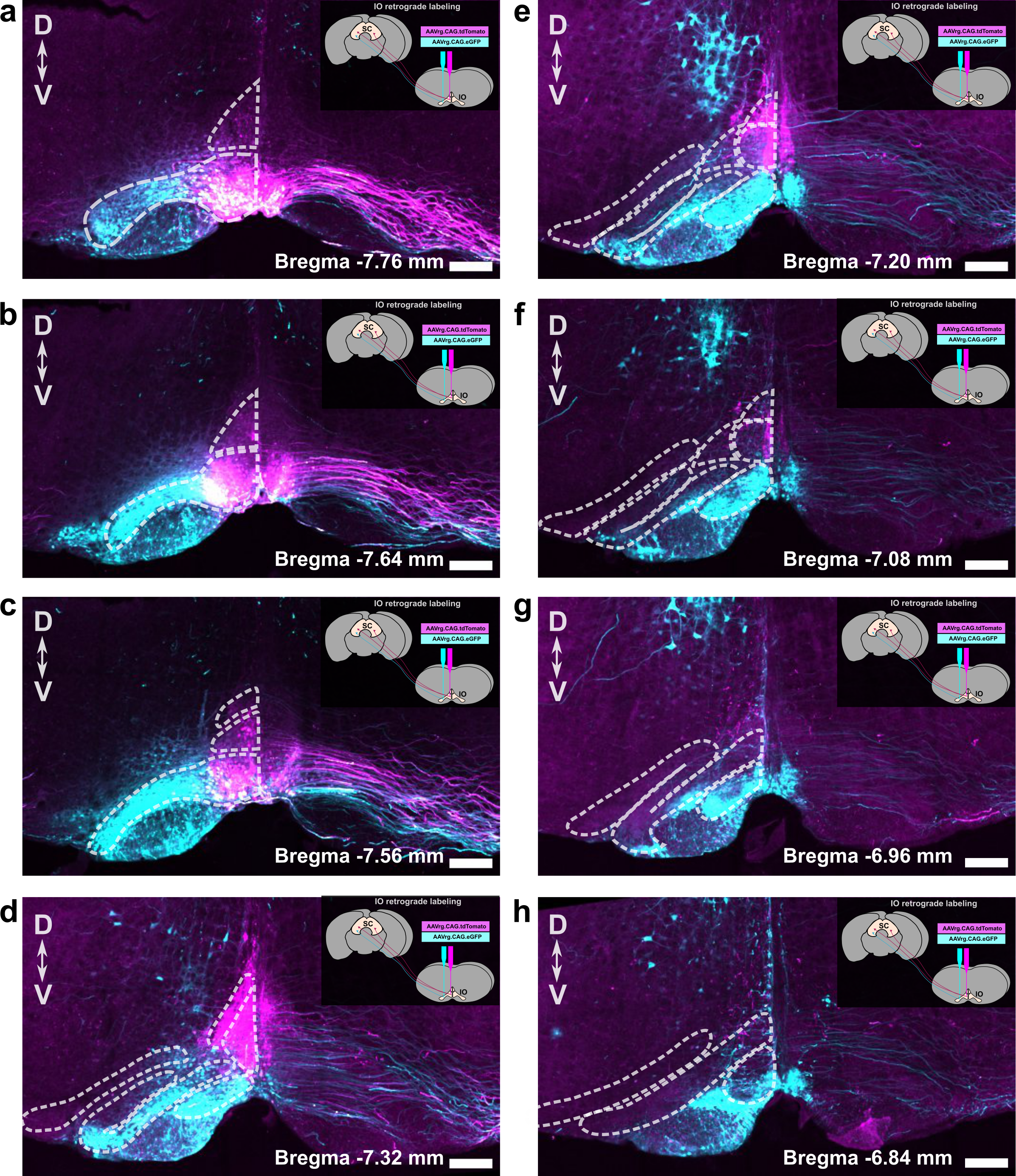

Supplement: Supplementary file 2 — (PNG 6917 KB) [file 429_2025_3032_MOESM2_ESM.png]

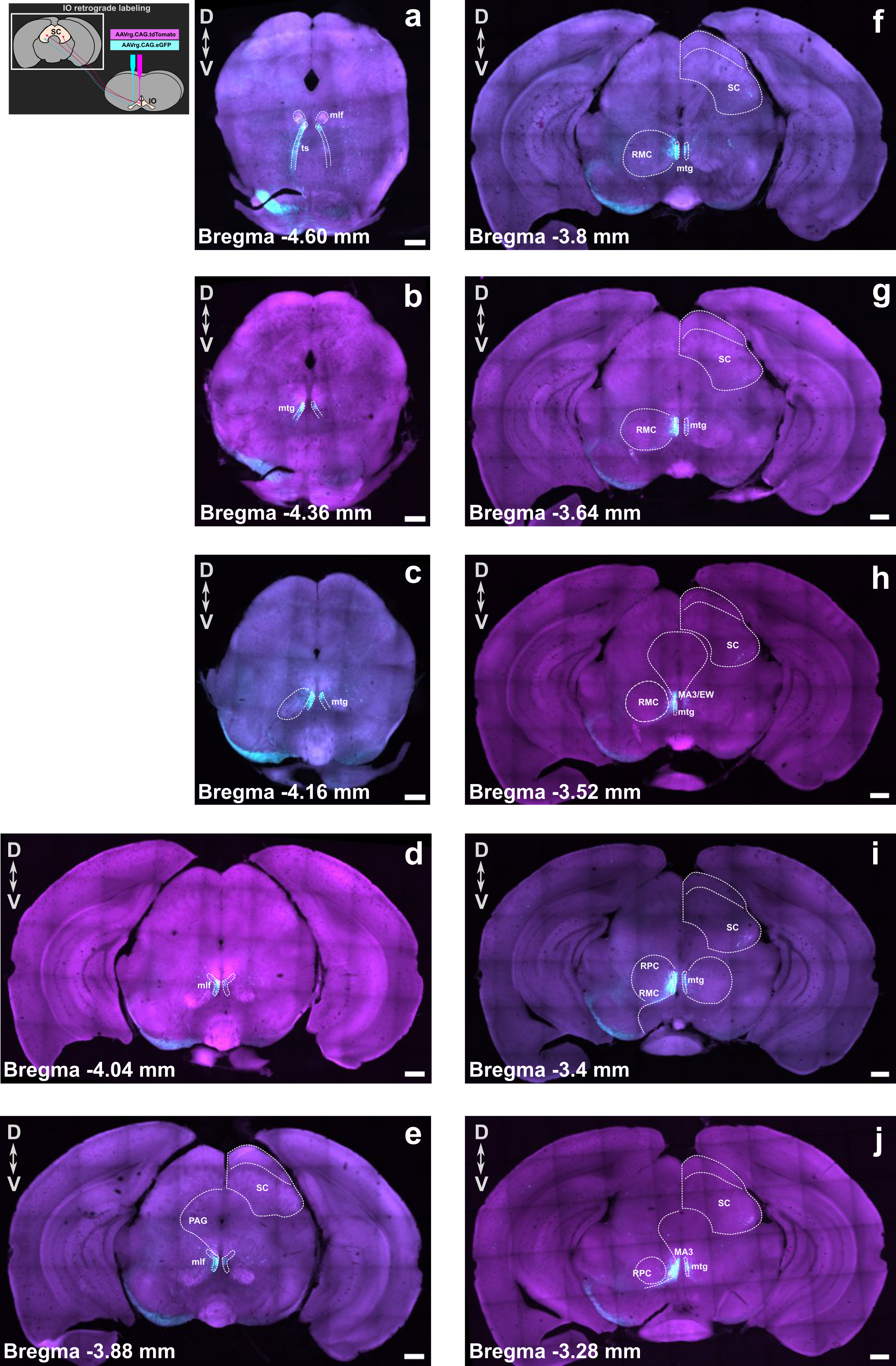

Supplement: Supplementary file 3 — (PNG 4755 KB) [file 429_2025_3032_MOESM3_ESM.png]

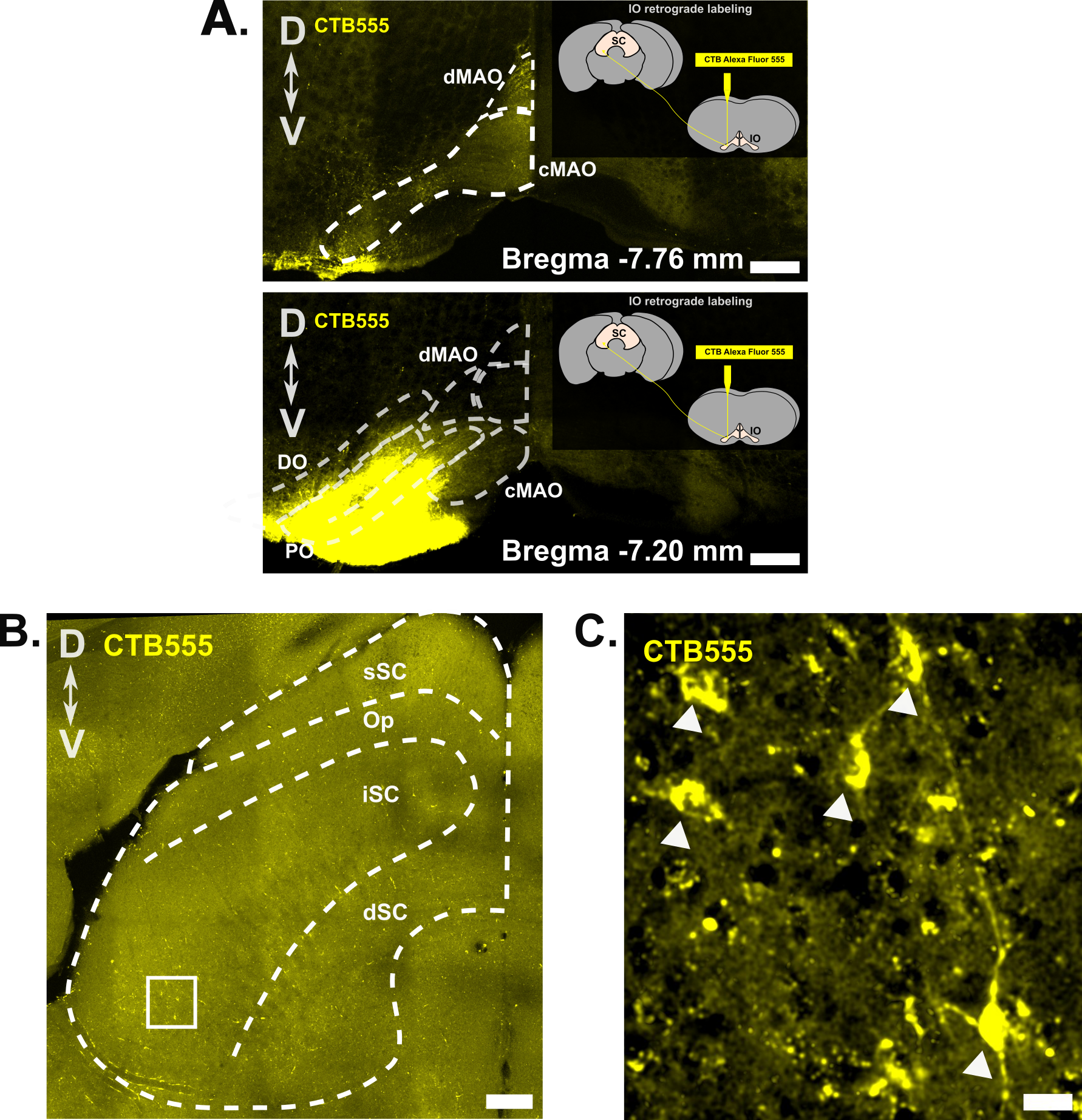

Supplement: Supplementary file 4 — (PNG 3368 KB) [file 429_2025_3032_MOESM4_ESM.png]
